# Monitoring, Modeling, and Statistical Analysis in Metal Additive Manufacturing: A Review

**DOI:** 10.3390/ma17235872

**Published:** 2024-11-29

**Authors:** Grant A. Johnson, Matthew M. Dolde, Jonathan T. Zaugg, Maria J. Quintana, Peter C. Collins

**Affiliations:** 1 Department of Materials Science and Engineering, Iowa State University, Ames, IA 50011, USA; grantajo@iastate.edu (G.A.J.); mdolde@iastate.edu (M.M.D.); jzaugg@iastate.edu (J.T.Z.); mariaqh@iastate.edu (M.J.Q.); 2 Ames National Laboratory, Ames, IA 50011, USA; 3 Center for Advanced Non-Ferrous Structural Alloys (CANFSA), USA; 4 Center for Smart Design and Manufacturing, Iowa State University, Ames, IA 50011, USA

**Keywords:** additive manufacturing, monitoring, modeling, statistics

## Abstract

Despite the significant advances made involving the additive manufacturing (AM) of metals, including those related to both materials and processes, challenges remain in regard to the rapid qualification and insertion of such materials into applications. In general, understanding the process–microstructure–property interrelationships is essential. To successfully understand these interrelationships on a process-by-process basis and exploit such knowledge in practice, leveraging monitoring, modeling, and statistical analysis is necessary. Monitoring allows for the identification and measurement of parameters and features associated with important physical processes that may vary spatially and temporally during the AM processes that will influence part properties, including spatial variations within a single part and part-to-part variability, and, ultimately, quality. Modeling allows for the prediction of physical processes, material states, and properties of future builds by creating material state abstractions that can then be tested or evolved virtually. Statistical analysis permits the data from monitoring to inform modeling, and vice versa, under the added consideration that physical measurements and mathematical abstractions contain uncertainties. Throughout this review, the feedstock, energy source, melt pool, defects, compositional distribution, microstructure, texture, residual stresses, and mechanical properties are examined from the points of view of monitoring, modeling, and statistical analysis. As with most active research subjects, there remain both possibilities and limitations, and these will be considered and discussed as appropriate.

## 1. Introduction

In the last couple of decades, the additive manufacturing of metallic materials has been the subject of significant research and investment, including considerable growth in the number of manufacturing systems available commercially and the development and growth of AM methods. There have been numerous research initiatives and activities adding to the available literature that have enabled the wider community to develop new understandings regarding the differences in processing and material state [1,2,3] between AM materials and traditionally manufactured ones (e.g., casting, welding, forging). Increasingly, new monitoring and computational modeling methods for AM processes, which permit the capture of qualitative and quantitative data as part of a “digital thread” for advanced manufacturing, are being developed and disseminated [4]. Table 1 and Table 2 show a summary of the state of the art of monitoring and modeling different variables and characteristics of AM parts.

Several alloys have dominated research activities and the industrial use of AM parts (e.g., titanium alloys, superalloys, aluminum alloys, refractory-based alloys, steels, and stainless steels) [5,6,7,8,9]. Given the governing thermophysical properties of the metals (e.g., melting point, boiling point, coefficient of thermal expansion, thermal conductivity, density, reflectivity), and their significant variability (e.g., thermal conductivity can range from <0.1 to ~4.0 W/cm·K), along with the details of the energy sources and deposition systems (e.g., wavelengths of lasers, beam profiling, scanning strategy), the various combinations of materials and deposition systems can either result in depositions that are “feasible” (i.e., high-quality builds) or “infeasible” [10,11]. If the alloying elements in a material system are incompatible with the processing physics, the parts can have high levels of defects, including defects that either appear during deposition or evolve/appear during post-deposition thermal treatments. In an extreme case, the build may not be possible to produce, experiencing material failures, such as delamination or cracking at length scales corresponding to the line-by-line or layer-by-layer processing length scales. Research continues to mitigate deleterious defects in materials with prints that are generally thought to be “infeasible” [12], thus enabling the AM of new and existing alloy systems; e.g., for some high-strength aluminum alloys that are susceptible to solidification cracking, the addition of inoculants has been successful in promoting homogeneous nucleation to limit solidification cracking [13]. Recognizing that deposition feasibility is an important concept, some researchers are directly studying feasibility over multidimensional compositional spaces, rapidly identifying interesting alloy pathways, and providing a key concept to enable future gradient materials [14].

**Figure 1:**
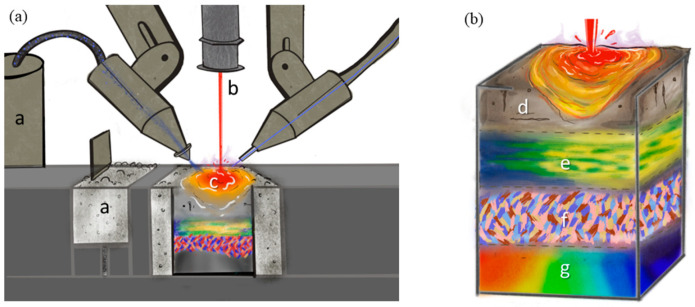
Schematic of an additive manufacturing process, separated into (**a**) processing and (**b**) material state, showing a—feedstock (powder, wire), b—energy source (laser or electron beam), c—melt pool, spattering and vapor plume, d—defects (spherical porosity, lack of fusion defects), e—compositional distribution, f—microstructure and texture, and g—residual stresses and distortion.

**Figure 2:**
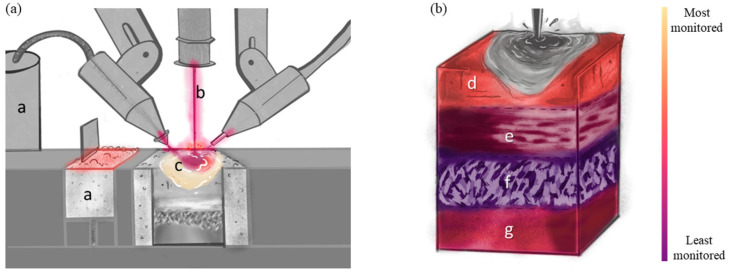
Schematic of an additive manufacturing process, separated into (**a**) processing and (**b**) material state, with an emphasis on monitoring, showing a—feedstock (powder, wire), b—energy source (laser or electron beam), c—melt pool, spattering and vapor plume, d—defects (spherical porosity, lack of fusion defects), e—compositional distribution, f—microstructure and texture, and g—residual stresses and distortion.

**Figure 3:**
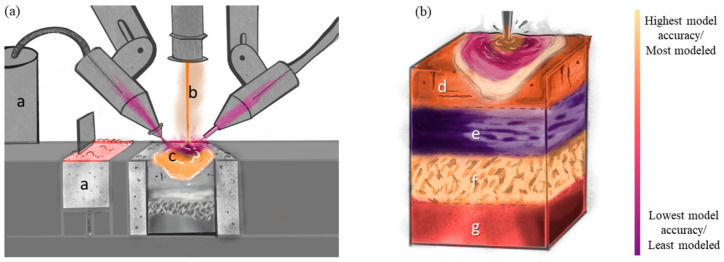
Schematic of an additive manufacturing process, separated into (**a**) processing and (**b**) material state, with an emphasis on modeling, showing a—feedstock (powder, wire), b—energy source (laser or electron beam), c—melt pool, spattering and vapor plume, d—defects (spherical porosity, lack of fusion defects), e—compositional distribution, f—microstructure and texture, and g—residual stresses and distortion.

**Figure 4:**
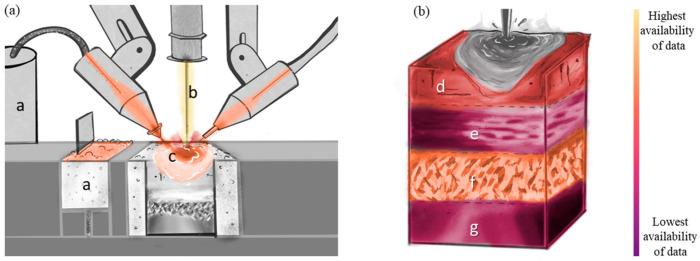
Schematic of an additive manufacturing process, separated into (**a**) processing and (**b**) material state, with an emphasis on statistics (or availability of data for statistical analysis), showing a—feedstock (powder, wire), b—energy source (laser or electron beam), c—melt pool, spattering and vapor plume, d—defects (spherical porosity, lack of fusion defects), e—compositional distribution, f—microstructure and texture, and g—residual stresses and distortion.

For this review paper, the monitoring, modeling, and statistical analysis methods for AM of metallic materials are classified according to two distinct perspectives, namely (i) the processing methods and (ii) the material states that are produced. Overviews of these perspectives are shown in Figure 1. The processing of AM (Figure 1a) encompasses a—the feedstock (i.e., powder, wire), b—the energy source (i.e., laser, electron beam, plasma arc), and c—the melt pool (including spattering and vapor plume). By changing any of these processing aspects, the final material state of the part (Figure 1b) will change accordingly. The material state includes d—defects, e—compositional distribution, f—microstructure and texture, and g—residual stress and distortion and their influence on mechanical properties. For monitoring, modeling, and statistical analysis, Figure 1 will be evaluated in depth individually. These evaluations will look like “heat maps” to elucidate perceived strengths and weaknesses on the same color scale—light to dark, respectively. For example, Figure 2 for monitoring considers the relative number of papers related to monitoring each aspect of processing and material state found in the literature. Figure 3 is similar to Figure 2, such that the color is relative to what has been modeled most and least frequently, while also taking into account the accuracy of the state-of-the-art models and simulations. Figure 4 evaluates the availability of data for each aspect of processing and material state, which can be captured through monitoring techniques and used to validate models and simulations.

In the context of this review paper, monitoring (Figure 2) is meant to imply real-time monitoring during the process, which can be difficult to define in a way that is either accepted broadly or applied easily. Of importance, we consider monitoring based upon the physics permitted and not any existing constraint of engineering systems; i.e., we consider what may be possible, rather than being limited to what exists. For the purposes of this review, the term “monitoring” is defined as (i) the use of measurement devices of varying modalities to (ii) obtain quantitative measurements that are spatially and temporally registered and that record (iii) interpretable signals of meaningful physical phenomena associated with the process and/or material state that (iv) significantly impact the quality of the material subsequent to the deposition and which, when aggregated, (v) have the potential to form a critical detail recorded in a digital thread (the term digital thread is one of two terms (the other term is a digital twin) that arises from the richness of the digital transformation associated with Industry 4.0. Digital twin refers to the concept of obtaining an exact replica of a part where, for each voxel (volume element), the manufacturing and material state attributes would be known and tied to a specific part—1:1. Digital thread refers to not only the lifetime of a part (i.e., the physical object is likely to deviate from the digital twin during service) but opens the door for statistical treatments of multiple parts). Such a digital thread could be used for quality control prior to placing a part into service, lifetime management for parts during service, or as information to support next-generation materials, processing, and design. Monitoring AM processes is important to ensure the quality of manufactured parts by rejecting those with an excessive number or type of defects (e.g., porosity, distortion, lack-of-fusion defects) before they are inserted into service and subjected to operational conditions (e.g., forces, stresses, temperatures) [10,15,16]. With the number of interdependent variables in AM, monitoring different features of the build process is a logical, though largely aspirational, goal.

Modeling and integrated computational materials engineering (ICME) is considered an increasingly important aspect of AM and is currently being used to predict the microstructure and properties of a part before building one [17,18,19,20,21,22,23]. Modeling (Figure 3) is a form of abstraction, and in the materials science discipline, it normally consists of mathematical relationships that are used to define how the material state reacts given specific processing conditions. Modeling how processing parameters influence the resulting material state and properties helps to ensure a proper understanding of the physics that exists during AM processing and that the manufactured parts have the desired properties.

Statistics have been used in the analysis of monitoring and modeling results to aid in interpreting correlations between processing and material state (Figure 4). By statistically analyzing these additively manufactured parts, manufacturers can have increased confidence that the parts they make are meeting or exceeding the specifications set by design or standards. In a survey, up to 47% of manufacturers reported that having a lack of confidence in the quality of additively manufactured parts was enough reason to not pursue this form of manufacturing [24]. The use of statistical methods and uncertainty quantification tools can help identify critical manufacturing signatures that are important to monitor and model. For processing, leveraging statistics helps ensure confidence that monitored data are both reasonable and representative. By statistically refining monitoring data, improving models to further understand the material state and resulting materials properties should be possible and result in increased confidence that the final parts are made as expected [25,26].

Important to all three components of this review paper (monitoring, modeling, and statistical analysis) are the concepts of length and time scales. The operating physical processes in the variably sized process zones span across length scales (10^−9^ to 10^−1^ m) and operate both during processing as well as following deposition (i.e., continued crack propagation in parts with high degrees of residual stress), and thus, time scales are also spanned (10^−8^ to 10^+7^ s).

This paper is organized under two broad primary sections: Section 2 is on processing, and Section 3 is on material state. Relevant subsections to each primary section, such as “Feedstock” (Section 2.1), are then described. Embedded in each subsection are details associated with the state-of-the-art in monitoring, modeling, and statistical analysis.

## 2. Processing in Additive Manufacturing

Additive manufacturing processes use computers to control the delivery of both the material feedstock and a quantity of energy into a position that is co-registered in time and space. Under the right conditions (e.g., sufficient energy density, thermophysical properties), the energy and feedstock combine to create a molten pool, whose interaction with the environment and realization of other complex physics results in the Material State (Section 3). Thus, in this section, we consider (Section 2.1) Feedstock and Environment; (Section 2.2) Energy Source and Thermal Distributions; and (Section 2.3) Melt Pool, which includes a discussion of the vapor plume as it is integrally coupled with the dynamics of the molten pool.

### 2.1. Feedstock and Environment

In fusion-based AM processes, such as powder bed fusion (PBF), powder-blown directed energy deposition (DED), and wire-fed based additive manufacturing approaches, including wire arc additive manufacturing (WAAM) and electron beam additive manufacturing (EBM), the feedstock is either powder or wire. This feedstock and base material then undergo highly localized fusion events (melting and solidification) that add material, volume by volume, to form the finished part (Figure 1a and Figure 2a). The PBF process starts by spreading a layer of powder onto a build plate (or a previous layer) to be selectively melted using either a laser or an electron beam as the energy source. Another AM process, DED, uses wire or powder that is fed into the focal point of a laser or electron beam.

While the objective regarding monitoring associated with this review paper is to focus on digital means of in situ monitoring, we will briefly note the classical methods of ex situ characterization and quantitative measurements of the starting material that will be used in the process. Classically, powder is characterized using a variety of techniques to assess its size, shape, density, and flowability. Typically, these techniques involve samples for optical or electron imaging, weight distributions using sieves, time-based techniques to determine mass flowrates, and geometrical measurements such as the angle of repose [27,28,29,30,31,32]. In both powder and wire, bulk chemical analysis and surface chemical analysis can be performed.

#### 2.1.1. Feedstock and Environmental Monitoring

The in situ monitoring of feedstock (Figure 2a) can be conducted but is not currently widely implemented. In principle, powder-based feedstock can be monitored using various methods. For example, one in situ monitoring technique uses optical techniques to image the powder during or following powder spreading [33]. These images can provide information regarding potential powder oxidation from differences in the color of the particles [34], cross-contamination also using color differences [35], uneven layer thickness using image intensity as a surrogate of a semi-insulating layer of variable thickness [36], and any machine issues or defects caused by the spreader [37] or anomalously large particles or other large debris. Recent work by Tan et al. [38] suggests that it may be possible to use principal component analysis to infer the quantitative signatures of conventional particle attributes (e.g., density, friction, interparticle forces) through measurements of the “avalanche” angle of powder against the powder spreader, which could be used as a continuous convolutional term of some of these fundamental metrics, typically measured externally in batches. Beyond these optical techniques, limited research has explored the use of eddy current measurements to monitor discontinuities in the feedstock layer [39].

Contrary to powder bed-based AM techniques, which provide spatially resolved information that could be used to correlate the powder to be processed with the process zone/material state, the monitoring of feedstock presents greater challenges for DED processes, as any correlation must be made through a time-resolved measurement of incoming material that is captured by the molten pool at some incremental time step later. Further complicating powder-blown DED techniques is the fact that only a fraction of the incoming powder is captured by the molten pool. Despite these challenges, researchers have demonstrated that the flow of powder can be monitored as it is blown into the melt pool using high-speed optical imaging systems [40]. Powder-based DED processes have the potential to record optical snapshots of powder within the powder feed system, including angles of repose or when the powder is entering the powder feed mechanism. Using such images, it would be possible to obtain information related to size, morphology, roughness, oxidation, or cross-contamination from limited analysis areas, i.e., the surfaces of volumes of powder [41]. Powder mass flow has also been studied using in-line acoustic measurements [42]. In certain materials systems and processing conditions where there is sufficient contrast for the modality of information in the images (e.g., light) and depth of information in the images, high-speed imaging of the powder flow approaching and interacting with the melt pool could have a sufficient resolution such that properties of the powder can be extracted (e.g., morphology, surface quality, particle size) [43]. The limitations include difficulties when the melt pool temperatures and emissivity prevent quantitative interpretable information from being collected.

While wire-based AM techniques do not enjoy as wide a variety of in situ monitoring techniques as powder-based systems, some techniques do exist. Optical monitoring of the wire can be conducted at the point where the wire feedstock enters the melt pool. Of particular interest to some researchers is the degree of wire deflection [44], as deflection is reported to be directly coupled with the tendency to form defects or achieve dimensionally accurate parts. However, beyond these techniques, there is limited literature available that describes the monitoring of wire feedstock for wire-based processes. The lack of research does not negate the importance of the subject. For example, a recent thesis by Ng Chi-Ho [45] demonstrated that the presence of surface contaminates (e.g., soaps used in wire drawing) could result in defects in additively manufactured builds, indicating that the chemical analysis of incoming wire could be an important parameter to monitor. Similarly, other researchers have indicated that internal defects within wires might lead to defects in the deposition. Looking forward, in this review, we suggest that an opportunity space exists to conduct research and development activities to monitor the wire using, for example, ultrasonic testing, optical imaging, instruments to monitor shape, and/or techniques capable of chemical analysis such as X-ray fluorescence (XRF) to detect composition anomalies.

In structural metals, the composition of the depositions is known to have a significant effect on the resulting microstructure, properties, and performance [46,47,48]. When AM approaches are adopted, the deposition composition will invariably differ from the “certificate of composition” that is associated with the material feedstock. The chemistry will change due to capture/alloying from contaminates or impurities carried by the feedstock (e.g., the soaps in the wire drawing mentioned previously), preferential evaporation [47], or ingestion of gases present in the chamber atmosphere (e.g., moisture, oxygen, nitrogen). Further, the atmospheric conditions may have an effect on material feed and build quality in other ways, such as altering powder flowability in powder bed systems, which can lead to predicting or inferring powder or part quality [49]. Thus, it is plausible that an important aspect would be to monitor the atmosphere within the build chamber and, if possible, the vapor plume (described in Section 2.3). Table 3 presents common monitoring techniques for each variable or characteristic discussed in this review, along with a rating for the amount of data collected for future use in modeling and statistical analysis.

#### 2.1.2. Feedstock Modeling

As with monitoring, the two most common AM feedstocks that are modeled are powder and wire (Figure 1a and Figure 3a). Approaches associated with wire modeling are often inherited partially from welding practices. Welding and AM share similar physics, so the overlap in concepts and models is understandable.

**Figure 5:**
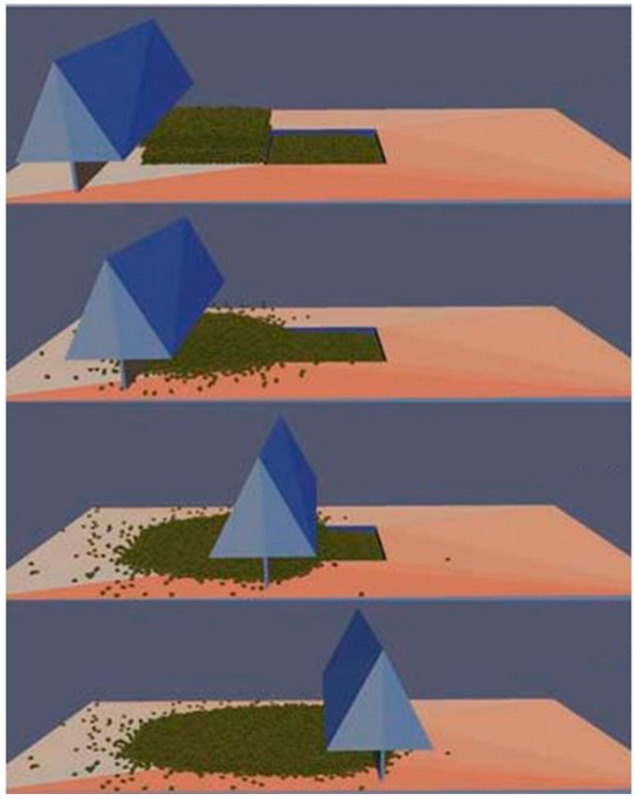
Discrete element model (DEM) simulation of additive manufacturing powder rake over time. Reprinted with permission from [141].

Arguably the most extensive effort, and thus most complete software that is available for certain aspects of powder modeling, is an extension of LAMMPS (Large-scale Atomic/Molecular Massively Parallel Simulator), which was created at Sandia National Laboratory [139]. The extension is known as LIGGGHTS (LAMMPS Improved for General Granular and Granular Heat Transfer Simulations) [140]. LIGGGHTS uses the discrete element method (DEM) to model the motion of particles [141] and is combined with computational fluid dynamics (CFD) to include the flow of surrounding liquid. This modeling technique gives the ability to model powder particle movement during raking and is a start to modeling complex interactions in the feedstock (Figure 5). DEM models will often use one of the following functions to describe contact mechanics: Hertzian, Johnson–Kendall–Roberts, or Derjaguin–Muller–Toporov [142,143]. The main difference between these models is how the cohesion is modeled. LIGGGHTS has been used to determine the powder flowability in DED [144]. LIGGGHTS has also been used to model how irregular powder can influence its flowability properties during AM [141], as well as modifying the surface finish during powder spreading in PBF [145]. In addition to LIGGGHTS, there are other software that have been developed for the purpose of modeling and simulating parts of the AM process and material state. These are shown in Table 4 in addition to common model formulations.

Wire AM techniques have also been modeled. However, this modeling has been primarily performed by the welding community. Adapting and modifying these models to AM may be possible for the techniques that use wire as feedstock. Many of the models related to wire techniques include more information related to the energy source; therefore, most of the models will be discussed later. However, as these models have matured over the years, more complicated and realistic models have been created. One example is a model of a twin-wire gas metal arc welding (GMAW) process [146]. For this process, two wires are used in the welding process. In welding, and therefore in AM, using two or more feedstocks is of some interest, especially in alloys that have significant changes in microstructure and properties with changes in feedstock to control local properties in AM (e.g., duplex stainless steels [147]), and thus, these additional model developments are of benefit to the wider AM community.

**Table 4:** List of modeling formulations and some software for AM.

Label	Description	Formulation	Software	References
a	Feedstock: powder feed, raking, and wire feeding	Discrete element method (DEM) with computational fluid dynamics (CFD); numerical models	LIGGGHTS	[140]
b	Energy source	Numerical models; finite element method (FEM) for deformation AM processes	N/A	[148,149,150]
c	Melt pool	FEM or finite volume method (FVM) CFD; material point method (MPM); numerical models; lattice Boltzmann method	AdditiveFOAM, ExaMPM, TruchasPBF	[151,152,153,154]
d	Defects	CFD; numerical models	N/A	[11,155]
e	Composition variation	Numerical models	N/A	[156,157,158]
f	Microstructure	Kinetic Monte Carlo (KMC); cellular automata (CA); phase field (PF); Potts model; CALPHAD Scheil-Gulliver model; numerical models	AMCAFE, AMPE, ExaCA, MEUMAPPS-SS	[14,159,160,161,162,163,164,165,166,167]
g	Residual stress	FEM	See Table 5	[22]

#### 2.1.3. Feedstock Statistics

Collecting, storing, and using the spatially- and temporally-rich data from process monitoring is computationally expensive. In an unconstrained processing environment, researchers might desire to store very large quantities of data and, as noted for the case of monitoring feedstock, could record high-speed optical images of all the powder entering the system (Figure 1a and Figure 4a). One can conceive that a sophisticated real-time image analysis program might then automatically detect size, morphology, and inhomogeneities. While this is lacking in the literature, and thus represents an aspirational possibility, once collected, it is necessary to consider the probabilities and statistics of such measurement [52]. For certain measurements, it is possible to leverage statistical descriptors that exist, such as particle size distribution (PSD) values of D10, D50, and D90 for the powder feedstock, where the designation (e.g., D10) signifies that 10% of the total powder is finer than the corresponding size (e.g., D10 of 15 µm indicates that 10% of the powder is finer than 15 µm). However, a single measurement of PSD for a well-mixed powder sample will invariably differ from the PSD for powder following a period of time where free-settling can occur [168] and thus will likely differ from the PSD of in situ measurements, where the integrated PSD over time (i.e., a PSD(t)) should approach measurements for a lot of powder. Similar time variabilities are expected for other property metrics, such as sphericity. However, in principle, it should be possible to develop a time-dependent model based upon powder flow, size, density, shape, free settling, etc., apply assumptions about the distributions of the model parameters (e.g., Gaussian or log-normal for powder size), and develop statistics associated with the dynamics of the feedstock [50,51]. To the authors’ awareness, such work has not been conducted for the dynamics of powder flow.

Another critical component to characterizing and analyzing the feedstock is absorptivity. Since factors such as the powder size distribution have an impact on absorptivity, direct measurement is difficult [169]. In a study concerning the measurements of absorptivity of metallic powder, two different PSD models were created, and changing the distribution from a Gaussian to a bimodal distribution made a significant difference in the measured absorptivity [24]. Further complicating the issue, the absorptivity of a single layer of spherical particles is higher than a flat surface, as incoming light can be scattered by the spheres followed by various interactions with other particles, effectively creating multiple scattering events. Therefore, if a piece of the as-built material or a small batch of the powder is taken aside for absorptivity testing, two different results could be obtained. This issue highlights just how important statistics are and how, if neglected or used inappropriately, the information gathered from monitoring or used in modeling can be markedly off from the ground truth.

### 2.2. Energy Source and Thermal Distributions

Common fusion-based AM processes use a laser beam, electron beam, or an electrical arc as energy sources (Figure 1a). The energy source is not only the main contributor to the overall heat input into the system but is also the one that is easiest to monitor and dynamically control as part of the process (the other heat source would be associated with enthalpies of reactions and phase transformations). The primary energy source is responsible for fusing the feedstock and enabling a stable melt pool. The accurate control of the incident power and shape of the energy source are important, as their control enables the process to be optimized, thereby increasing the repeatability of the process and the attending properties of the additively manufactured components.

Similar to feedstock materials, considering the classical approaches to measuring attributes of the energy source is useful. Regarding lasers, their power at various points along a beam path can be measured using a thermopile. Such data can be used to develop calibration curves, confirm incident power prior to/following depositions, or diagnose the “health” of AM systems. Similarly, certain operators may wish to record the profile of their beam prior to and/or after depositions. In such instances, beam profilers can be used. Regarding electron beams, their power can be either calculated using the settings or measured more accurately using, for example, a Faraday cup. The latter is preferred when aspects of the electron guns, such as apertures, are subject to evolution/drift/degradation over time.

#### 2.2.1. Energy Source Monitoring

By using certain optical components, including particular configurations of beam splitters and/or partially reflective mirrors, collecting laser power dynamically during a deposition is possible (Figure 2a). Under certain manufacturing conditions, operators may wish to monitor the shape of a beam and could adopt beam splitters or partially reflective mirrors to monitor the shape of the incident beam. Published research on direct energy source power monitoring is limited or non-existent for laser and electron beams. Measurements in electron beams mostly fall in the category of voltage differences between the electron source and the grounded build plate [170] or currents in lenses that can be correlated with the positions of the beam. Research on monitoring the arc in wire arc processes is far more mature since the technology is derived from welding. Multi-sensor setups to monitor multiple parameters of the arc are currently used in laboratory systems [54] as well as in commercial systems [55]. These setups involve monitoring the voltage, current, sound, radio frequency, and temperature of the arc [54]. Acoustic monitoring during AM builds has shown to be effective for defect detection in multiple systems, but experimentation to detect other processing parameters such as laser power has been explored only in laboratory settings [53].

While monitoring the energy source can provide quantitative information regarding the energy entering the process zone, only a fraction of the incident energy is available to the material to undergo the necessary thermophysical processes (e.g., fusion for liquid-based AM) to achieve the desired material state. The general categories of energy loss are known and include (i) energies that are lost through inefficiencies of “coupling” between the energy source and the material, such as reflection and scattering from the incoming material and/or scattering/absorption in a plume above the substrate; and (ii) energies that are lost due to traditional heat transfer balances, including conduction, convection, radiation, and the thermodynamics of phase transformations. The heat flow during an AM build can influence the final microstructure and properties [171,172]; thus, from the perspective of the material state, it is arguably more important to conduct in situ monitoring of the temperature distributions (e.g., gradients, time-dependent heat flows) than the energy of the system. In situ monitoring approaches for heat flow include the use of thermocouples [56,172] to monitor the temperature at specific locations over a period of time. Thermocouple-based temperature monitoring can take place at the build plate [57], close-to-the-line depositions [58], or even within a deposition by inserting (i.e., “harpooning” [173]) the thermocouple into the melt pool or directly depositing on top of the thermocouple [174]. Infrared (IR) imaging and pyrometers can measure the temperature of the current layer as well as previous sections of the build [59,60]. Monitoring the build temperature for PBF can be accomplished after the rake of a layer of new powder and can help estimate the layer quality while also monitoring the temperature at specific areas [36]. Table 3 presents common monitoring techniques for each variable or characteristic discussed in this review, along with a rating for the amount of data collected for future use in modeling and statistical analysis.

#### 2.2.2. Thermal Modeling

The complex thermal gyrations associated with AM that exist as a result of the repetitive motion of the energy source relative to previous built material represents a unique challenge when seeking to understand and describe a thermal history, which has an effect on the material state and therefore the properties. There are several methods that are used to model the spatially and temporally dependent thermal histories, ranging from simple heat source models (Figure 3a) to more complex finite element method (FEM) models (Table 4) [175].

The first models for the heat flow of a moving heat source in welding were presented by Rosenthal [148]. These equations provided a starting point for refinement by several researchers in the welding community over many years [149]. Other numerical models have been developed for many types of arc welding methods, including gas tungsten arc welding (GTAW) [176] and plasma arc welding [177]. However, many of these models can be modified and used for new applications, such as different AM processes.

Heat source models are often used in conjunction with mechanical models to optimize welding parameters for different alloys and predict distortion [178,179]. For example, Chen et al. [150] used CFD and finite volume method (FVM) models to predict the weld pool and thermal dynamics during welding, which further enabled the prediction of optimized weld parameters such as arc current and speed for different thicknesses and materials.

In these multi-physics models, one of the key parameters is the functional forms of the energy distributions, which will vary by the nature of the energy source. Several common energy distributions exist, such as Gaussian, top hat, and inverse Gaussian. The shape of the laser can influence the solidification structure of the alloy by changing the thermal gradients, interfacial velocities, and thermal gyrations [180].

#### 2.2.3. Energy Source Statistics

**Figure 6:**
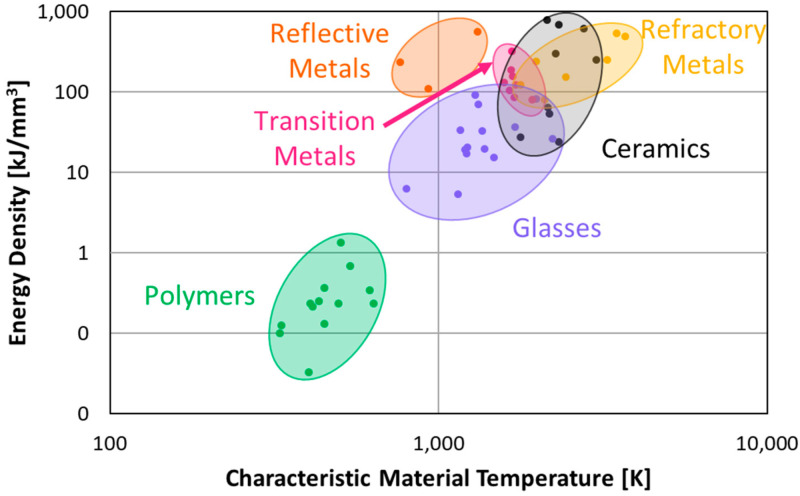
Ashby-like diagram of volumetric energy density versus characteristic material temperature for various material types categorized by color. Recreated with permission from [181].

Monitoring aspects of the energy source is critical to understanding the conditions the material experiences, but as energy sources vary from system to system, the actual data record may seem not to be transferrable from machine to machine, a problem that can be overcome using a combination of dimensional and statistical analysis [61]. To describe the energy source more accurately between machines, the energy density is used [63,64,65], which is a convolution of the power of the energy source and the volume of the melt pool (Figure 4). Figure 6 shows a general relationship between the characteristic material temperature and the energy density required for manufacturing each material. This figure was developed by collecting experimental details from more than 100 papers across all types of additive manufacturing processes and materials classes. As the reader might surmise, few of the papers from which these data have been extracted present their experimental settings as energy densities. However, each contained sufficient details regarding exposure time (e.g., speed or time), energy input, and volume processed to extract a nominal energy density. The authors recognize the exceptional variability that such an analysis of research papers incorporates. However, despite the uncertainty, it is noteworthy that this correlation is not only valid for metal-based AM but seems to be general for all variants of additive manufacturing. This general trend provides a good first step for what processing variables should be chosen to additively manufacture different materials, regardless of the system used. However, it is possible to extend beyond this simple relationship by, for example, performing a dimensional analysis on the processing inputs that can further abstract the AM process. An example of this approach as applied to SLM has been demonstrated in [61].

Convoluting energy source variables such as power, shape, and speed can result in higher-order dimensionless variables. By listing all the process variables in a system and systematically combining them into new dimensionless input variables, the total input variables needed will decrease (by the total amount of fundamental dimensions in the system) [61]. By representing the inputs in this new way, the number of supposed process variables needing to be tested to provide a whole picture can be decreased. Without dimensional analysis, two research groups with different source power limits would not be able to replicate experiments between them effectively. However, by manipulating other dimensional process variables, the two groups demonstrated that they could effectively circumvent this power difference by achieving similar dimensionless variables by now having the same system [62]. This would allow for a research lab with an EBPBF and another lab with an LPBF system; while having completely different energy sources, they could still maintain similar build conditions by targeting similar higher-order variables such as energy density, even though their process variables are quite different.

Another method for optimizing process parameters involves the creation of code surrogates [182]. As opposed to running full simulations, these code surrogates are both faster and less complex than simulations and thus are ideal for rapid and broad testing to identify regions of viability and not necessarily completely replace simulations. A case study evaluated using code surrogates in AM [182]. From a small data set, the most promising surrogate code, a Gaussian process (GP) code surrogate, was able to predict the region of viable energy densities that most closely matched the region of viable energy densities made from a much larger data set [182]. Using this GP code surrogate could be a beneficial approach to finding a region of viable process parameters, cutting down computation time, and allowing researchers to obtain samples faster.

### 2.3. Melt Pool

Fusion-based AM depends on a heat source melting the feedstock and forming a molten pool of material (Figure 1a) that solidifies upon cooling. However, prior to solidification, there are physical attributes and dynamics associated with the melt pool that are of interest to those who seek to control the process and material state. Among the most important physical parameters and dynamics are (i) the physical shape of the molten pool, including both what lies above the deposition plane that is governed by wetting and what lies below the deposition plane that is governed by heat transfer, thermal gradients, and interface motion; (ii) dynamics associated with a keyhole (if present), including keyhole collapse; and (iii) dynamics associated with convection and the capture/retention of defects within the liquid.

Notably, not all of these attributes and/or dynamics are quantifiable using existing techniques. Even classical measurement techniques, such as the measurement of wetting angle or velocity of solid–liquid interfaces, are often limited to analogs [183,184,185] rather than the direct measurement of the materials of interest. Some limited ex situ techniques can be used for the direct measurement of the melt pool. Optical or electron imaging can detect the melt pool size and shape depending on the alloy.

#### 2.3.1. Melt Pool and Vapor Plume Monitoring

**Figure 7:**
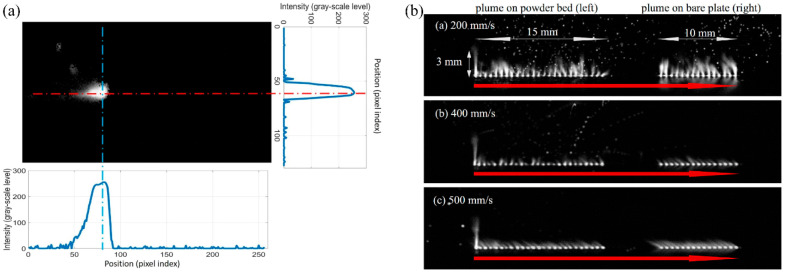
Examples of monitoring melt pool and vapor plume. (**a**) Vision-based in situ monitoring results of melt pool detection in LBPF, (**b**) effect of scan speed on vapor plume in LBPF, the scan direction is indicated by the red arrow. Figure 7a is reprinted from [66] under Creative Commons Attribution License (CC BY); Figure 7b is reprinted from [188] under Creative Commons Attribution License (CC BY).

Whereas for feedstock and energy sources, brief discussions of existing measurement techniques are merited, there is little “classical” work on melt pool size. Thus, there exists a robust body of modern and emerging peer-reviewed work on melt pool monitoring (Figure 2a and Figure 7 and Table 3) in the literature, as it represents an area of active research by many groups [66,71,81,98,186,187,188]. Not only do many commercial AM systems have melt pool detection and monitoring systems [189], but research groups have developed and integrated their own monitoring techniques into a wide variety of systems. Optical monitoring is the most common approach for both laboratory and commercial systems, as it can be deployed using low-cost complementary metal oxide semiconductor (CMOS) cameras mounted coaxially or off-axis [66,67]. An analysis of optically monitoring the melt pool can include detection methods to separate the melt pool from solidified material, spatter (or small amounts of liquid material ejected from the melt pool), and other sources of optical emissions [66,76]. As the data sets are large, new approaches for automated image processes and quantitative analysis range from simple pixel intensity segmentation [190] to machine learning or neural networks for the automatic detection of melt pool boundaries [68]. In addition to the low-cost CMOS camera detectors, high-speed imaging is also used for melt pool monitoring in multiple laboratory systems [191], although there is a concurrent increase in the size of the data sets and a decrease in ease/speed of data processing.

Thermal and IR-based sensing techniques are also widely explored to conduct melt pool monitoring. Thermal imaging is used in both on- and off-axis monitoring configurations, identical to optical imaging [69,70]. Melt pool size determination is based on the liquidus-solidus transition point and determining if the temperature of a particular pixel or cluster is above or below this transition point [71]. Thermal monitoring data can be used to train machine learning models that are subsequently used to simulate melt pool properties and automatic liquid–solid boundary detection models, as will be discussed later [72]. A limitation associated with thermal and IR sensors is the accuracy of the calibrations to relate the intensity of a pixel cluster with known transition temperatures (e.g., solidus, liquidus). There is also the potential complication of metal vapor plume gases coating optics or other monitoring components [113]. Along with traditional thermal imaging that measures surface temperature during the AM process, emerging work on the use of X-ray radiography to monitor the sub-surface temperatures and melt pool dimensions during deposition has been conducted [192].

X-ray-based monitoring techniques are emerging in the AM space. These techniques allow for the real-time viewing of phenomena such as powder mixing within the melt pool [74], the liquid–solid boundary [193], and the evolution of spatter [73]. X-ray backscatter detection can also be used for monitoring the melt pool as well as the plume and spatter during a deposition [75]. However, these methods are limited by the depth of the X-ray, the resolution desired, the richness (size) of the data, and the geometry of the parts being deposited. Thus, these X-ray-based techniques more widely provide in situ data to better understand the governing physics and develop accurate models rather than necessarily providing real-time in situ process monitoring.

Methods other than optical, thermal, and X-ray-based imaging for monitoring the melt pool of AM processes are not as well studied but can still be useful. Ultrasonic measurements can detect melt pool dimensions due to the sharp decrease in the shear modulus and density in the liquid phase [77]. Eddy currents and radio frequency emissions have also been studied for melt pool monitoring [75].

During fusion-based AM processes, after melting the metallic feedstock (both powder and wire), the material continues to heat, and if the evaporation temperature is reached or exceeded, evaporation occurs, forming a vapor plume [194]. Continued heating can cause the metal vapor to become plasma and be added to the plume [194]. The infrared imaging of the plume in PBF processes has been used to support the development of statistical studies to detect melt pool instabilities [78]. Optical imaging is also a popular technique for monitoring the vapor plume during a build and has been studied in multiple systems [79,80,81]. The plume, consisting of vaporized metal from the melt pool (Figure 7b) [188], provides a physically relevant foundation upon which other advanced monitoring techniques for the chemical composition of the build have been developed, including, notably, laser-induced breakdown spectroscopy techniques (LIBS). This monitoring method has been used for detecting specific elements during laser-based AM builds [82,83,84,85]. Schlieren imaging is an optical imaging technique that can monitor density gradients and flow in gases. Schlieren imaging can be used to monitor the shielding gas flow as well as processing by-products and evaporation during the AM process [195]. Optical emission spectroscopy is a similar technique for monitoring the vapor above a deposition that can be used for monitoring the melt pool, surface, and subsurface conditions [86].

Spatter in AM is the ejection of material from the fusion zone. Spatter can consist of liquid droplets ejected from the melt pool or unmelted feedstock that is blown away from the melt pool zone [87,196]. Optical monitoring with image thresholding to classify spatter during a build has been carried out in multiple systems [81,87,88]. Optical imaging often uses image processing to determine the location of the spatter. The optical imaging of spatter patterns can be used to calibrate models to predict spatter locations and distributions in future builds [91]. X-ray imaging can give higher resolution monitoring of the spatter, as the optical emissions of the melt pool and plume do not need to be segmented out from the image for the analysis [197]. X-ray-based spatter monitoring has been conducted in multiple laboratory AM setups [89,90]. Optical imaging is also used to monitor spatter patterns to validate models [91].

Many of these monitoring techniques, including optical and thermal imaging, can be integrated and optimized using techniques such as machine learning, genetic algorithms, artificial intelligence, and others to aid in the analysis of the large amounts of data generated during monitoring, reduce the time required for such analysis, and provide predicted information for future decisions. One example is a study using a deep learning-based approach to create a model to analyze the melt pool in an LPBF system [198]. Other authors have reportedly used integrated imaging combined with a neural network to monitor the melt pool either using optical or thermal methods in DED systems [199,200].

#### 2.3.2. Melt Pool Modeling

**Figure 8:**
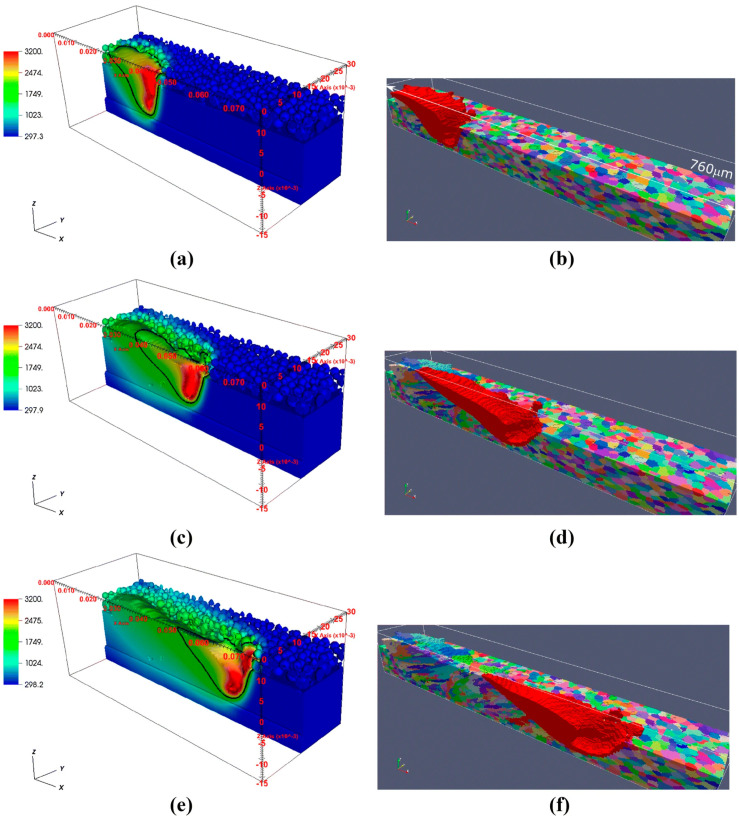
Integrated thermal profile (**left column**) and solidification (**right column**) model at 327 μs (**a**,**b**), 674 μs (**c**,**d**), and 967 μs (**e**,**f**). Reprinted with permission from [18].

As stated earlier, there have been significant advancements in the understanding of melt pools in AM via in situ studies, including X-ray imaging of thin sheets [201,202], which have provided essential information for melt pool modeling (Figure 3a and Figure 8) [18]. Several methods and software packages exist that can model melt pools (Table 4), and they often use FEM or FVM for CFD, e.g., Flow-3D [203]. Flow-3D, a commercially available software package, can model and simulate melt pools to elucidate the influence of processing parameters on defects [155]. Other models for melt pools include smoothed particle hydrodynamics (SPH) [204], which can also be used to help with defect modeling [155]. FEM and similar modeling methods are particularly well suited for heat flow because of energy source–material interactions and are commonly used to simulate and study the complex thermal histories associated with AM processes [205,206,207,208,209].

ExaAM, an effort of the U.S. Department of Energy’s Exascale Computing Project (ECP), has created several software packages for AM modeling [210]. The models and software packages that have been created are intended to be integrated together, as seen in Figure 8, which is an integrated thermal and solidification simulation. A few software packages from ExaAM are especially well-suited to simulate and study the melt pool, including AdditiveFOAM, Truchas/TruchasPBF, and ExaMPM [151,152,153,211]. AdditiveFOAM is built on OpenFOAM [154] and uses CFD to calculate fluid flow and heat transfer in the melt pool and to simulate the solidification but does not include microstructural evolution and is limited to the liquid-to-solid phase transformation. Similarly, Truchas, made for casting solidification simulations, and its powder bed fusion counterpart TruchasPBF use FVM CFD to model thermal histories in AM. The thermal histories simulated in AdditiveFOAM and TruchasPBF can then be used to simulate grain structures through an ICME framework when connected to a microstructure model. This will be discussed in Section 3.3. Another interesting bit of code that has been developed by the ECP project is ExaMPM. ExaMPM uses the material point method (MPM), a variant of the particle-in-cell (PIC) method [153]. ExaMPM is able to resolve the physics and dynamics associated with the complex interactions found in melt pools, including those between and among solid, liquid, vapor, and powder. One exciting possibility is the inclusion of interactions between the laser and matter. This software is intended to be able to model many of the complex interfaces found in AM melt pools (Table 4).

As mentioned previously, the interaction of the energy source with the feedstock and substrate material can often lead to dynamic and competitive phenomena, such as vaporization in a plume or ejection of molten material, known as spatter. Since these phenomena are difficult to model and simulate directly, creating simplified models that ignore certain details of the physics to ascertain the result rather than the process is useful [157,212]. One example of a simplification of complicated physics in the energy source–feedstock interaction is the use of the Langmuir equation [156] to describe vaporization and gettering (solute loss and pickup) [19,213,214]. Models of metal constituent vaporization can be tied to thermal models to estimate material and solute loss in AM builds [158]. Other events that result from the dynamic nature of the melt pool in AM include the formation of a keyhole, as well as material ejection [215,216,217]. These events can lead to chemical differences in the build as a result of process variations [218,219].

#### 2.3.3. Melt Pool Statistics

The use of statistics to analyze aspects of AM processes, such as the melt pool, can involve both the quantification of the uncertainties associated with monitoring data, as well as the validation of models (Figure 4a) [92,93,94]. Sensitivity analyses consider the various sources of uncertainty that accumulate from a model and how these various sources contribute to the overall uncertainty. Sensitivity analyses of differing AM processes have shown that small changes to machine settings have a considerable effect on the overall result [220]. With the use of thermal cameras to monitor the size of melt pools, sensitivity analyses can inform on which parameters vary the size of the melt pool, which in turn can help validate models [95].

Understanding which elements may preferentially vaporize during an AM process, as well as their relative rates of loss, enables the prediction of the composition of the final build. One study [214] created a model of how much aluminum would evaporate during the Ti-6Al-4V build. Sensitivity analysis was completed on this model as a first step to ensure the total error in model predictions was not greater than the expected results. This was a novel and better approach than was standard at the time, but the model had to be validated with the previous evaporation of aluminum in titanium data. Using a monitoring technique such as LIBS to measure vaporized elements in the plume of the sample could better inform these models, though few researchers have attempted to integrate LIBS directly into an AM system. Further sensitivity analyses could be conducted to understand how varying the process variables (and by extension the melt pool) can influence preferential evaporation.

Gathering statistics from spatter has been accomplished through monitoring the plume [221]. Using a support vector machine or SVM (a form of supervised learning algorithm), variations of the plume frame to frame from an inline infrared camera are tracked. When large variations are present, the material is likely in an unstable state (e.g., spattering, keyholing). By training this SVM on the monitored data from earlier in the build on whether the spatter has occurred, the model can predict not only when the spatter occurs but also the potential creation of defects if the spatter lands back on a susceptible surface. One interesting example of the interplay between monitoring, modeling, and statistics is exemplified by new research into the use of electromagnetic techniques to monitor the plume and spatter. By aggregating spatter data from cameras and considering particle statistics, a new model has been developed, supporting the theory that new instrumentation can be deployed.

One emerging technology that leverages statistical methods is the detection of spatter in real time. A maximum-entropy double-threshold image processing algorithm that is based on a genetic algorithm (MEDTIA-GA) has been used to recognize spatter from monitoring data, and its results were compared to three other traditional threshold segmentation methods (Otsu’s method, the triangle threshold segmentation algorithm, and K-means clustering algorithm) [222]. The processing time (and thus the computational overhead) of this novel GA approach was, at worst, an order of magnitude lower than these other methods. The MEDTIA-GA method has also demonstrated an ability to not succumb to segmentation errors such as noise sensitivity, spatter conglutination, and spatter omission [222]. Emerging statistical methods like these are instrumental to both improving confidence in obtained data and making statistical analysis more approachable by reducing computational costs.

## 3. Material State of Additively Manufactured Materials

The term “material state” of a component is related to the concept of material state awareness (MSA), which is defined as the “digitally enabled reliable nondestructive quantitative materials/damage characterization regardless of scale” [1,2,3]. From a traditional materials science perspective, this includes but is not limited to the following attributes: composition, solute distributions, microstructure (phases, their size, distribution, and correlations), crystallographic texture, and the presence of defect structures (e.g., dislocations, porosity, interfaces, cracks) across all length scales and couples to more macroscopic non-traditional materials science attributes, such as surface roughness and the shape/topology of the part or component. Each of these attributes (both microscopic and macroscopic) is informed in part or completely by the processing and can have large effects on the properties and performance of the material.

**Figure 9:**
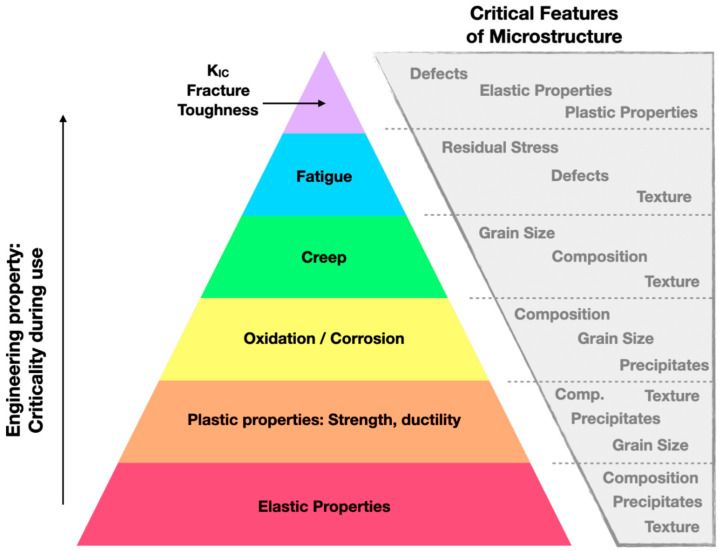
Hierarchy of mechanical properties with associated critical microstructural features (based on Maslow’s hierarchy of needs [223]).

It is worth briefly considering how these aspects of the material state influence the properties of the material. Here (Figure 9), we introduce properties in a manner somewhat analogous to Maslow’s hierarchy of needs [223], where, as we proceed from the most foundational design properties (e.g., elasticity and plasticity) to the properties that become critical when materials are put into service (e.g., fracture, fatigue), we correlate them with the most critical aspects of the microstructural state.

From this type of hierarchical map, we can begin to understand the process-structure-property correlations. For example, additive manufacturing can produce a wide variety of defects depending on the processing parameters. Defects can result from extremes in the energy source (e.g., laser power that is either too low or too high), which cause abnormalities in the melt pool, such as keyholing and balling [11,155,224]. Excessive residual stresses originate from the complex thermal gyrations and can cause cracking [206,225,226,227] either during the build or during relaxation in periods of time (e.g., weeks) following deposition and can have a deleterious effect on many critical mechanical properties, including ductility and fatigue [15,228], even though strength may be increased due to Taylor hardening [138]. Further, residual stresses can lead to distortion and can be very spatially dependent due to complex thermal histories [225,227,229]. While it is tempting to assert that the composition of the part is set by (or equivalent to) the certified composition of the feedstock, through previous discussions in this paper, it is emphatically noted that since AM is a dynamic process, extreme processing conditions can change the composition locally or globally. These compositional changes can further influence the local and overall properties of the material [219]. The microstructure can also have a marked effect on the properties of a material. Given that the microstructure of any arbitrary material is strongly influenced by its thermal history, it follows that the energy source and melt pool represent the most important corresponding processing parameters in AM [20,210,230]. Collectively, these interrelated material state attributes govern the properties (discrete measurements) and performance (statistical distributions of properties) of the material. Therefore, while the mechanical properties are not normally included in the material state, they have been added to this section in this review. Therefore, monitoring and modeling AM builds is important while considering and analyzing the data created statistically to understand how the material state develops during AM and what the resulting material state will be.

### 3.1. Defects

As with most engineered materials (except, for example, materials with engineered porosity levels), the presence of defects is generally considered to be undesirable, attributing to a general reduction in many properties including mechanical, electrical, and thermal. The AM process can result in a variety of defects, some of which are not found in materials produced using other processes. The defects commonly found in AM (Figure 1b) include porosity, lack-of-fusion defects, delamination, cracking, and balling [231].

Defects within AM can be detected and measured post-deposition through both destructive and non-destructive evaluation (NDE) techniques. Common measurement methods include X-ray imaging, ultrasonic testing, and microscopy [232]. Standards exist for some types of flaw characterizations and techniques for detecting them using NDE, including X-ray computed tomography, eddy currents, acoustic emission, and X-ray backscatter [233]. Classic metallographic characterization is useful for evaluating some types of defects such as lack-of-fusion (LOF) defects, spherical porosity, and hot cracking. Depending on the length scale, these defects are detectable and may be quantitatively measured using either optical or electron imaging techniques.

#### 3.1.1. Defect Monitoring

Monitoring defects within AM is, quite appropriately, an active area of research, as their presence can greatly diminish the properties of the final part (Figure 2b). Monitoring and detecting aspects of these defects (e.g., location, size, type) during a build and developing strategies for the mitigation/elimination/repair of defects are important aspects of ensuring part quality. Setups that are common for in situ process monitoring techniques of a build are similar to those for defect detection within the material state. The coaxial imaging of the melt pool can map porosity volume and location using a trained convolutional neural network, which can then be confirmed using X-ray computed tomography (CT) [96]. High-speed optical signatures during a build can be correlated with ex situ characterization of defects [97]. Optical imaging has been used to monitor balling [98] and detect spatter and holes in a PBF build [99]. Optical emission spectroscopy has been used in some studies for porosity monitoring [100].

Defects including porosity, LOF defects, delamination, and cracking all include either a separation of the layers in a build or the build not being fully dense. As the thermal conductivity of these voids, whether internally under vacuum or entrapped gas, is significantly lower than the surrounding material, measurable differences in the thermal signatures are observed. Thermal imaging has been successful in detecting defects by showing discontinuity in thermal signatures [234,235]. Multiple sensor setups have been developed using a combination of optical and thermal imaging techniques for the purpose of monitoring builds for defect detection [236].

**Figure 10:**
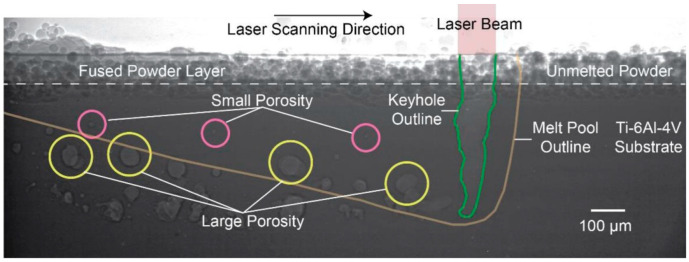
In situ X-ray imaging showing defect formation and melt pool outline detection in LPBF. Figure is reprinted from [237] under Creative Commons Attribution License (CC BY).

The use of X-ray imaging for defect monitoring is an emerging detection method that typically uses X-rays produced from a synchrotron. While using a synchrotron is not feasible for most commercial applications and systems, as the samples need to be very thin, research has been conducted using this technique to show the physics associated with the formation of different types of defects, such as keyholing or entrapment in low-temperature regions of the molten pool (Figure 10) [237]. The high-speed X-ray imaging of the keyhole threshold and morphology has been studied [101]. Research into keyhole porosity monitoring using a synchrotron has shown ground truth observations with high resolution [102].

Spattering can cause defects in a build such as not fully reintegrating to the melt pool, creating uneven thickness for subsequent layers, and changing the composition of the part, as the spatter can have a high oxygen content [90]. Spatter monitoring for the purpose of defect detection has been successful using high-speed optical imaging during a PBF process [103].

As mentioned in Section 2.3, techniques such as machine learning, genetic algorithms, and artificial intelligence have aided the analysis of melt pools during AM processing. In addition, these techniques have aided in the detection of defects and certain characteristics such as origin, size, and morphology. One study captured and processed high-speed optical imaging, including the signatures of the spatter. Another example is the MEDTIA-GA algorithm, mentioned in Section 2.3.3, which was used to automatically detect spatter signatures [222]. Other examples include machine learning combined with artificial neural networks [238], convolutional neural networks [239], support vector machines [240], and tree algorithms [241] to detect defects during printing. Multi-sensor setups have been shown in LPBF systems to identify defect formation using a deep convolution neural network (CNN) [242]. Monitoring the bead dimensions of a deposition can be paired with neural network models such as a dimensionless artificial neural network (DI-ANN) to be used for defect monitoring in a DED system [243].

Acoustic monitoring, a common non-destructive evaluation technique, uses a sensor to pick up generated elastic waves in a material. Acoustic monitoring setups have been shown in multiple laboratory settings. A study using acoustic monitoring has shown success in detecting LOF defects within a build along with high-speed imaging and photodiode data [244]. Acoustic monitoring of defect events during a build was performed and then correlated to the type of defect [104]. For example, acoustic signatures from cracks have been monitored successfully during a build [105]. Table 3 presents common monitoring technique defects.

#### 3.1.2. Modeling Defect Formation

**Figure 11:**
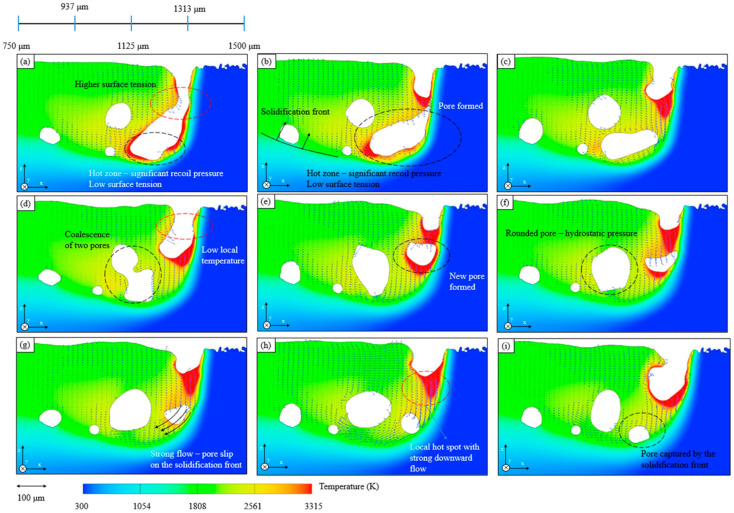
Finite volume method (FVM) model of melt pool with keyholing and pore formation over time (**a**) t = 2.395 ms, (**b**) t = 2.4 ms, (**c**) t = 2.415 ms, (**d**) t = 2.43 ms, (**e**) t = 2.445 ms, (**f**) t = 2.45 ms, (**g**) t = 2.455, (**h**) t = 2.47 ms, and (**i**) t = 2.495 ms. Figure is reprinted from [155] under Creative Commons Attribution License (CC BY).

The most common defects within metal AM builds are LOF defects, keyholing, and spherical pores. Keyholing can be avoided by ensuring the combination of the power source and speed (and thermophysical properties of the material) are such that the energy density does not lead to excessive vaporization [224]. However, energy densities that are too low can also result in the generation of other defects [107]. Within a single AM process such as LPBF, even the laser scanning strategy can change the amount of spherical defects present [218]. Due to the thermal physics and fluid dynamics of the melt pool, as well as the thermal gradients and viscosity of the liquid, pores can become trapped. Spot scan strategies have differing melt pool morphologies, which result in the retention of fewer spherical defects. Thus, modeling the physics of the melt pool is inherently important to the prediction of porosity (Figure 3b and Figure 11) [155], as the thermal gradient present (driving force for pore movement by convective flows) is outweighed by the drag, resulting in pores that are not able to rise to the surface and be eliminated. Such modeling has been conducted with a moderate degree of success [245] by abstracting the shape of the melt pool to have one “depth” parameter, but due to the three-dimensional morphology of melt pools, using more complex shape parameters, as well as including diffusion of heat from previous passes, can produce results that are closer to reality [224].

#### 3.1.3. Defect Statistics

The statistics associated with the defects (Figure 4b), including the conditions under which they form, their formation frequency, and their geometric metrics (i.e., size, proximity, and location), are necessary to predict the performance of any given part [63]. Considering the potential quantity of parts that could be manufactured for industrial application, proper statistical treatments enable the systematic study and implementation of strategies to reduce/eliminate defects, design topologies/shapes to meet the expected service demands (e.g., mechanical loads) [106], or even classify parts as “acceptable” or “reject” based upon monitoring signatures, especially once paired with machine learning algorithms [239]. These methods are necessary due to the variability that metal additive manufacturing is subjected to. For instance, one case study looked at the repeatability of an SLM printing process and found it to be acceptable under the guidelines of engineering standards for dimensional and geometrical analysis [246]. However, a large-scale industrial study [247] of manufactured tensile samples found that nearly two percent of samples, though nominally geometrically similar, failed catastrophically. These failures were correlated with parts in which there were clusters of LOF defects whose configuration significantly degraded the structural integrity of the test coupons. The Beese Research Group [248,249,250,251] has published pioneering research that incorporates full stress triaxiality and the Lode angle parameter to develop models to understand how defects (and defect clusters) interact with the design/topology of the part to understand deformation and failure. Regarding in situ quality control, the probability of some attribute of defect formation (e.g., size, number, location) can be correlated with whether an anomalously poor property might be expected, and thus, the part should be categorized as a failed part prior to use [245,252,253]. After studying the concentration of defects within a build process, a probabilistic simulation, such as a Monte Carlo simulation, could be run to determine the percentage of parts that match differing thresholds for the probability of success/failure [224].

Notably, such statistical approaches can incorporate data from modeling, measurements, or a hybrid of both data types. For example, in situ monitoring in combination with a machine learning algorithm could be used to set thresholds for differing variables such as defect size, location, and the density of the defects being detected. This has been demonstrated by optimizing the energy density of prints to minimize the formation of defects such as pores, lack of fusion defects, and keyholes [254,255,256]. This combination could also statistically determine when enough defects are in proximity to one another for catastrophic failure to occur during printing or during operation.

### 3.2. Compositional Distribution

In metals and alloys, the chemical composition has a very significant effect on the mechanical, electrical, thermal, and physical properties. Thermal processing and solidification can lead to chemical segregation within a part [253]. Further, in additive manufacturing, different elements have different propensities to evaporation (or gettering), leading to selective loss (or gain) of elemental species, resulting in compositions (Figure 1b) in the build that differ from the feedstock input [257].

Classic ex situ approaches to measuring the composition of AM samples include energy dispersive spectroscopy (EDS), X-ray fluorescence (XRF), inductive coupled plasma (ICP), or X-ray diffraction (XRD). These techniques are often limited to some degree. For example, EDS, XRD, and ICP are often destructive. Similarly, EDS and XRD require particular details to be met for each specimen, whether it is a polished surface (EDS) or size/flatness (XRD).

#### 3.2.1. Elemental Monitoring

**Figure 12:**
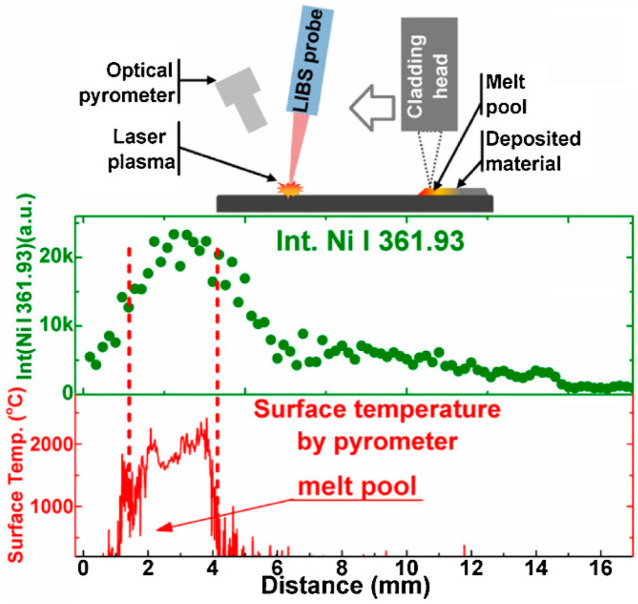
Schematic of laser-induced breakdown spectroscopy (LIBS) to optimize data collection of composition and temperature. Figure is reprinted from [82] under Creative Commons Attribution License (CC BY).

The elevated temperatures in the melt pool can cause substantial metal vaporization [217]. Monitoring chemistry then becomes important for understanding and predicting the compositional distribution in the build during printing (Figure 2b, Table 3). As mentioned previously, LIBS is a characterization method based on detecting a light spectrum of metallic vapor (Figure 12) [82]. A study using a custom in situ LIBS setup monitored varying compositions of WC in a NiFeBSi matrix [82]. This study showed direct LIBS measurements at the melt pool as well as post-deposition trailing for the cladding head on the hot but solidified metal. Optical emission spectroscopy (OES) is similar to LIBS where the vapor is monitored and different light wavelengths are calibrated to different elements, but in traditional LIBS, the solid is directly ablated to vapor, while OES can be calibrated to monitor the metal going from the solid to liquid phase (melting) and liquid to solid phase (evaporation) [110]. For example, the vaporized metal composition has been monitored using laser-induced plasma emission spectroscopy during a Ti-Al binary AM build, showing the Al content at different locations [84]. Using OES, the preliminary results showed that differences in composition in a Ni-based alloy could be measured [110]. Other research involving OES monitoring was conducted in a Cr and tool steel system [258].

Although the most promising in situ monitoring techniques for compositional analysis are based on LIBS or OES, alternative technologies have been explored. For example, researchers have demonstrated that a mass spectroscopy-based technique can measure the ejecta and atmosphere near the melt pool [108]. Using custom setups, energy-dispersive XRF has been shown to monitor Cr and Ni in the vapor produced during an LPBF process [109]. In the future, AM processes using an electron beam could use existing electron microscopic techniques, including either an in situ EDS detector or a backscatter detector to monitor the composition of the deposition with either a wide area averaging or spatially resolving compositional fluctuations within the build. Some other characterization techniques such as acoustic or ultrasonic testing could be applied to in situ monitoring setups if the composition varied enough to influence the elastic properties of the alloy.

#### 3.2.2. Modeling Composition

As O’Donnell et al. [219] showed, processing parameters can lead to changes in the local or overall composition of an AM build. Therefore, understanding how the energy source and processing parameters influence the composition is important. As stated in Section 2.3, there have been efforts to understand vaporization and gettering using the Langmuir equation [19,156,213,214]. While this equation has been used to identify and understand trends in compositional changes based on thermal histories [158], it has not been applied to understand how processing parameters can influence the local composition of a build (Figure 3b). The thermal [259,260], solidification [219,261], and mechanical properties [219,262] of a material can change with changing composition. Understanding and modeling compositional changes as a result of the processing parameters may improve the accuracy of future models of AM parts that seek to understand local microstructure and properties.

#### 3.2.3. Statistics of LIBS Data

Accurately identifying the composition of local areas within a part is inherently useful to ensure the part is built to specification [111,112]. As mentioned, the primary in situ monitoring method used to obtain this data is LIBS. To analyze LIBS data in real time, however, spectra from the samples need to be generated, and characteristic peaks for differing elements need to be found and deconvolved, which can be nontrivial. Similar confidence intervals could be generated, as was mentioned for feedstock analysis, but another method could be implemented to reduce the computation time of deconvolving peaks. Using prior data of spectra from both the pure elements, as well as binary or higher-component alloy standards, independent regions of each element can be flagged [263]. The intensities associated with the spectra of the pure elements can be compared to the intensities of the standards, permitting the creation of a function that maps spectral signatures with the composition of the measured specimens. Further, a machine learning algorithm might be developed to ingest real-time LIBS data, then use this heuristic to not only analyze spectra faster but also provide information regarding statistics of specimens over builds. With even minimal updates to this methodology or computational power, this would be close to achieving real-time analytical speeds that would allow the full compositional distribution of data throughout the build process (Figure 4b).

### 3.3. Microstructure and Texture

The microstructure of metal parts made using fusion-based AM processes can be vastly different from traditionally manufactured materials and can vary significantly between different processes. The microstructure is often set by the solidification rate and the thermal gradient, which are governed by the equation $R=\left(1/G\right)\left(\partial T/\partial t\right)$, where R is the solidification velocity, G is the thermal gradient, and ∂T/∂t is the cooling rate. The microstructure can also evolve over time after solidification due to phase transformations and heat accumulation.

Measuring and characterizing the microstructure of AM samples is commonplace. Optical and electron microscopy, XRD, and EBSD are all methods used to image and measure the microstructure of a sample post-deposition. Sample preparation techniques are important for all of these methods in order to ensure accurate results. Microstructural measurements can include the phases present, fraction of the phases present, grain or feature size, grain or feature geometry, and texture (Figure 1b).

#### 3.3.1. Microstructural Monitoring

The microstructure of an AM build governs the material properties [138,264,265,266,267]. Owing to various reasons (e.g., practicality, time, chemical handling, technological limitations), most monitoring techniques cannot directly observe the microstructure of a build (Figure 2b, Table 3). In addition to the most obvious limitations associated with implementing “traditional characterization” in situ—for example, the difficulties of polishing, chemically etching, and imaging a microstructure to mimic optical microscopy—any microstructure that could be revealed from a surface characterization technique may not necessarily correspond to the microstructure at that precise location following the completion of an arbitrary build. Indeed, microstructures are known to evolve due to remelting and subsequent thermomechanical gyrations, phase transformations, and microstructural evolutions. Despite these difficulties, there exists a strong base of knowledge from the casting and welding communities, as well as arising from computational efforts, to provide the materials scientist guidelines to set their process parameters to affect particular material states. For example, for the thermal gradient ($G=\left|\nabla T\right|$) and solidification rate ($R=\left(1/G\right)\left(\partial T/\partial t\right))$, interfacial velocity can be used to predictably set processing conditions to either promote columnar grains exhibiting texture or equiaxed grains without texture [268,269,270,271]. Even with this knowledge, the aforementioned issues lead to most monitoring techniques targeted at observing the microstructure using indirect measurements to classify the microstructure of an in situ build. Thermal imaging has been used in a few laboratory setups to observe the build and calibrate thermal data to microstructural formation and evolution. For example, in an electron beam PBF system, IR thermal imaging was used successfully to observe the build and the thermal gradients and match the IR intensity data to the microstructure at different locations in the final part [113]. A system using a combination of sensors including an IR camera was able to monitor melt pool temperature, real-time cooling rates, and thermal maps and then, using this data, was able to create a closed-loop system to control the microstructure by controlling the cooling rate of the deposition [114]. Similar approaches have been used for general microstructural details such as grain size based on melt pool size and other thermal characteristics of an AM build [115,118]. Ultrasonic inspection techniques enable the inference of details of microstructures, such as an assessment of the phases present, their fraction, grain sizes, and whether the crystallographic texture is present, as differences in each result in attending variations in the measured signals, and, when anisotropy is present, result in directionally dependent responses, including in the wave attenuations [116,117]; however, acoustic waves are generally interpreted in a more general or bulk manner rather than a site-specific manner. Perhaps the most direct method is an emerging approach based upon spatially resolved acoustic spectroscopy (SRAS) [131,132,133,134], which has been applied to the surfaces of additively manufactured specimens, demonstrating that it is possible to spatially map texture from as-deposited surfaces [272]. Similarly, for hybrid variants of AM, there is a possibility to use force feedback machining to map grains [135,136,137], though there are several engineering challenges to deploying this at the time of this paper.

#### 3.3.2. Modeling the Material State

The execution of complex models of different AM processes requires multiple iterations of calculations at a wide range of time or length scales, from predicting local thermal history to simulating the effects of residual stress. However, errors and uncertainties can transfer and compound rapidly between each of a series of integrated computations. Therefore, a methodology to quantify the error in models and ensure the models’ validity is necessary. A group has created such a process as a part of the Exascale Additive Manufacturing project (ExaAM) [273] (Table 4). Upon modeling the deposition of an AM sample using their Frontier Exascale Computer, select regions were chosen to predict their properties, and experimental samples were made to compare to the simulation results. After running their uncertainty quantification algorithm, they predicted that their compounded error should not be statistically significant, which, if correct, would help validate their model. Comparing experimental data to their simulations showed that they correctly predicted that the error was not statistically significant. While this scale of computing remains limited to only a few machines, their computing power makes quantifying errors in models possible, and as technology continues to mature, the accessibility by more researchers to such machines should increase. Further, once a limited number of highly complex simulations are executed, it is possible to develop and use simpler, surrogate approaches. While new models are being developed constantly, standardizing how the data are created and stored is important. One way that this can be conducted is by using the software DREAM3D. Created as a data pipeline to support the reconstruction of serial-section experiments in the earliest days of 3D materials characterization, DREAM3D expanded into software that may be used to build synthetic microstructures, generate surface meshes, and help with microstructural quantification [274]. There are other methods that store microstructural data, such as electron backscatter diffraction (EBSD) data and the statistical analysis of grains and their size and texture. There is extensive software available for visualizing EBSD data, such as the MTEX toolbox for MATLAB [275].

Regarding the foundational details of AM microstructures, there is much to build upon, as primary solidification microstructures have been studied for many decades (Figure 3b). There is a solid basis for knowledge related to solidification structures and their relation to processing parameters for traditional manufacturing methods such as casting and welding. A portion of this knowledge can be readily applied to AM. For example, one crucial aspect that determines the microstructure, as mentioned in the Monitoring subsection, is the G/R ratio. At low G/R ratios, equiaxed grain growth is often observed, whereas at high G/R ratios, columnar grain growth is found [167,276]. The G/R ratio can change the overall microstructure of a part, as seen by Dehoff et al. [271]. Solidification speeds can also change the microstructure within layers, known as the columnar to equiaxed transition (CET). The CET is a phenomenon where the solidification structure starts as columnar growth and ends as epitaxial growth, forming equiaxed grains [277]. Another important factor that governs the microstructure is the thermodynamic stability of phases. Some common AM alloys are inherently two-phase alloys, such as α/β titanium alloys and nickel superalloys [16,138]. Other alloys can form deleterious phases at intermediate temperatures, such as stainless steels [278]. Therefore, it is common to see CALPHAD and solidification models, such as Scheil-Gulliver models, intertwined with other microstructural models to serve as a basis for how a microstructure evolves in a dynamic process [14,279].

There are several ways that the solidification and microstructure of AM can be modeled. The most common types of models are those based upon kinetic Monte Carlo (KMC), cellular automata (CA), or phase field approaches (PF) (Figure 8) [18,175,280]. In general, PF has the best ability to connect to thermodynamics and kinetics in real life but is also the most computationally intensive of the three models. KMC uses randomness to model microstructural growth and is not very computationally intensive but is hard to connect back to physical representations. CA offers a compromise on both fronts, as it can connect to real life through its model formulation and is often not as costly as PF models. This compromise is the reason that CA has become one of the most popular methods for solidification modeling in recent years [165,281,282,283]. Texture is commonly modeled using CA and PF for AM, with recent introductions of texture modeling with KMC [175]. As with many models, it is common to see experimental validation of models that are created and reiteration of experiments with data that is gleaned from simulations. One example is the modeling, simulation, and experimental validation of a Ti-xW binary alloy and its solidification structure [284,285].

Several codes have been created to model and simulate AM microstructures from ExaAM. These microstructure codes are ExaCA, AMPE, Tusas, and MEUMAPPS-SS. These codes use a variety of model types and have unique microstructural aspects that they are designed for. ExaCA is a CA code that simulates grain growth [160]. CA is a relatively simple model to implement and has relatively low computational cost using one of several mathematical formulations, such as finite element (FE) and finite difference (FD) [175]. AMPE and Tusas are PF codes that are designed for simulating subgrain microstructural features during solidification by solving systems of partial differential equations [161,162,163]. MEUMAPPS-SS (Microstructure Evolution Using Massively Parallel Phase-field Simulation for Solid State) is a PF code written originally in Fortran that was converted into C++ and focuses on simulating solid-state transformations that occur as a result of the complex thermal histories seen in AM [164]. The US Naval Research Laboratory (NRL) has also created AMCAFE, a CAFE (Cellular Automata Finite Element) model to simulate the solidification in AM [165]. The model has been validated using a laser PBF 316L build.

Kinetic Monte Carlo microstructure models have also been made for AM [159]. SPPARKS (Stochastic Parallel PARticle Kinetic Simulator) was created at Sandia National Laboratories to pioneer a way to model microstructure. SPPARKS has modules specifically for AM applications and uses the spin-based Potts model by introducing probabilities to change the spin [286,287]. While KMC can simulate texture, SPPARKS cannot. As AM parts are highly textured, leading to anisotropic properties [288], the modeling and simulation of texture formation and evolution becomes important. Of the above codes that are mentioned, ExaCA and AMCAFE also are capable of simulating texture in AM. However, there is ongoing research to extend texture modeling to more methods, such as Monte Carlo (MC). For example, Pauza et al. [166] used an MC Potts model to model texture in additive manufacturing using Inconel 718 [165].

#### 3.3.3. Microstructural Statistics

Predicting the microstructure of any as-built part relies on having detailed knowledge of relevant aspects of the selected AM process conditions, as well as the material used [111]. An aspirational goal is to integrate monitoring data and modeling to predict local microstructure, its evolution, and, consequently, local and global properties, and performance (Figure 4b). Such a goal has been realized to different degrees of success in which researchers have integrated thermal models and physical processes such as evaporation and microstructural evolution to predict microstructure and have used sensor data to calibrate the models [115,213,289,290]. An example of a more fully integrated workflow is the ExaAM project that has produced multiple different models targeted towards metal AM processes, one of which is aimed towards predicting local microstructure; however, the problem that exists in all models, i.e., verification, remains reliant on the use of statistics, thus requiring additional computational resources. One open area of potential research is the use of statistical methods to understand the uncertainty and fidelity of difference monitoring approaches when implemented into AM systems.

### 3.4. Residual Stresses and Distortion

Residual stresses and distortion are inherent in AM processes (Figure 1b). The complex thermal histories that exist spatially and temporally within and among layers can lead to complex stress states. Distortion is a direct cause of these residual stresses, leading to the deformation of the material from the intended shape of a part due to the thermal stresses that exist during the manufacturing process.

Classic approaches to residual stress measurements use techniques such as XRD, neutron diffraction, ultrasonic techniques, and measuring relaxed strain through the hole drilling method [291]. Traditional measurement techniques use a powdered sample or a polished surface on bulk material or are semi-destructive to the sample. Depending on the level of stress within a sample, measuring geometric features or final dimensions can show distortion.

#### 3.4.1. Monitoring of Distortion and Residual Stress

**Figure 13:**
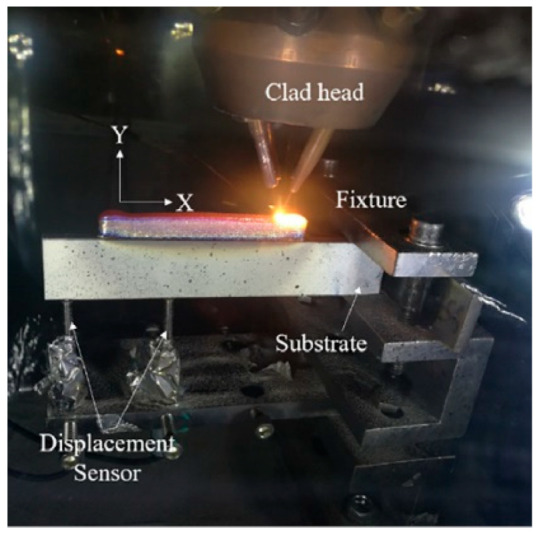
Experimental distortion detection setup used in a laser AM setup. Figure is reprinted from [120] under Creative Commons Attribution License (CC BY).

The line-by-line, layer-by-layer nature of metallic-based AM necessarily means that the material experiences gyrating thermal gradients and cyclic heating and cooling. Such gyrating thermal gradients and cyclic thermal cycles result in complex residual stresses that impact the mechanical properties of the final part (Figure 2b), such as strength and fatigue life, and can lead to cracking or distortion [225,226,229]. Efforts to minimize or control residual stresses in AM usually involve preventing steep temperature gradients by preheating the build/deposition/powder bed or applying different scan strategies [225]. Residual stress can be characterized using both destructive and NDE techniques. Some NDE techniques involve diffraction-based methods such as XRD, by measuring beam broadening that indicates lattice distortion of the crystal structure of the material and determining dilation in any direction. However, the need for a perfect stress-free control sample to obtain baseline lattice spacing is difficult during an AM process leading to diffraction-based methods being less suitable than other residual stress monitoring techniques, such as ultrasonic measurements [127]. Ultrasonic monitoring includes a variety of methods in multiple systems [119] and is viewed by many as a more viable approach to monitoring residual stresses than diffraction-based methods. If the residual stresses are large enough, the part can experience significant distortion, and under extreme cases can fracture or fail while still attached to a build plate. This correlation between distortion and residual stress provides a basis for monitoring distortion using approaches such as digital image correlation (DIC) [292]. Distortion measurements can use single-point laser displacement sensors (LDS) or full-field (2D or 3D DIC) imaging to monitor the distortion of the build or build substrate (Figure 13) [120,121,122,128]. Distortion measurements based on DIC use multiple points on a sample, tracking their relative displacements to provide distortion information in at least two dimensions, though more advanced 3D systems have been used to monitor the build plate during steel cladding tests [123]. Further, a system has been developed to show the full-field displacement of a laser cladding system using a partially fixed build substrate allowing distortion [124]. Distortion has been evaluated, as each new layer has been deposited [125]. Importantly, the natural inconsistencies on the surface of a DED build were used to show the full-field strain of a thin wall [126], as opposed to the typical DIC approaches, which require the application of artificial, stochastic speckle patterns. For monitoring, residual stress distortion-based systems are more widely studied and are the most feasible of the current sensing technologies (Table 3).

#### 3.4.2. Modeling and Understanding Residual Stress and Distortion

There are a multitude of reasons to model and predict distortion and residual stress, ranging from (i) the desire to produce a part in as near to net shape as possible, (ii) the desire to avoid post-deposition stress relief [293] treatments, and (iii) the need to mitigate/eliminate risks associated with sudden fracture/failure events.

**Figure 14:**
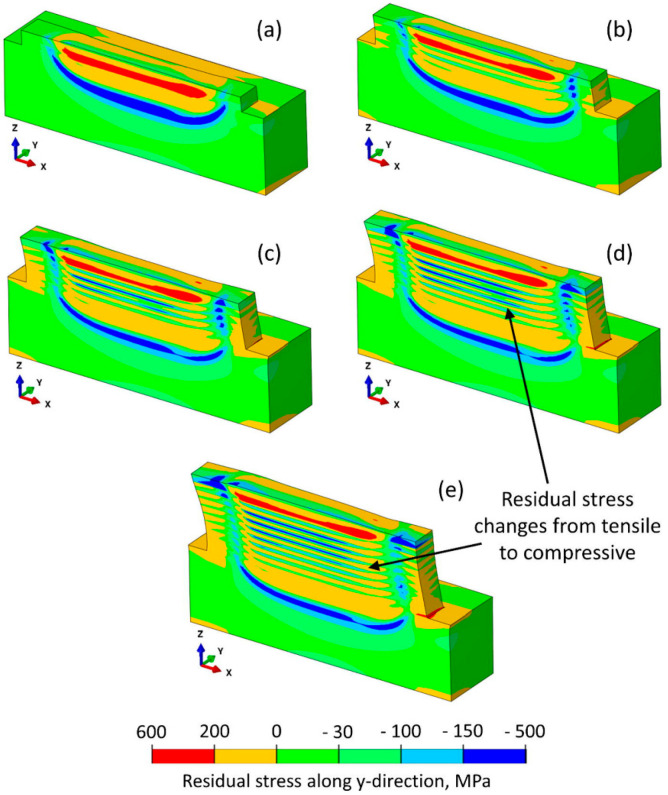
Modeling of residual stress in an additively manufactured sample at different layers. Figure is reprinted from [227] under Creative Commons Attribution License (CC BY).

There has been significant research in the last decade to create models that predict residual stress and distortion (Figure 3b and Figure 14) [227]. FEM is commonly used to model residual stress and distortion due to its common use for mechanical models. Since residual stress is a result of the large thermal gradients in AM, models require thermal information derived from heat source models [128,179,294], which were discussed in Section 2.2 [225,226,227,229,295]. Over time, these models have developed more complexity, considering phase transformations that can negate significant amounts of residual stress [178,296]. There are several commercially available software packages that estimate the residual stress and distortion of AM parts (Table 5) [297,298,299,300,301,302,303,304].

**Table 5:** Commercially available AM modeling software packages in alphabetic order.

Name	Company	Modeled Features	References
**3DEXPERIENCE**	Dassault	Thermal profile and stresses	[297]
**Additive Print**	ANSYS	Distortion and residual stress	[298]
**Amphyon**	Oqton	Thermal profiles, distortion, and residual stress	[299]
**COMSOL** **Multiphysics**	COMSOL	Coupled thermomechanical models	[300]
**GENOA 3DP**	AlphaSTAR	Defects and distortion	[301]
**Netfabb**	Autodesk	Thermal profiles and distortion	[302]
**Simufact** **Additive**	Hexagon	Thermal profiles, distortion, and residual stress	[303]
**Sunata**	Atlas 3D	Thermal profiles and distortion	[304]

#### 3.4.3. Statistics of Distortion and Failure Prediction

The statistical approaches available for predicting distortion are very immature (Figure 4b). Such immaturity is due, in large part, to the nature of distortion and its strong dependence on not only process parameters and thermal gradients but also how those thermal gradients couple with the part topology through the full 3D stress and strain tensors [129,296,305]. It is essential to recall that residual stress is not an average singular value but depends upon lattice distortion gradients in the object. Thus, residual stress is not even directly tied to dislocation densities, though this is erroneously assumed at times. A part can have a very high dislocation density (and thus strength) but have few if any gradients in the dislocation densities. Conversely, steep gradients can exist in parts with lower average dislocation densities. Thus, residual stress is the descriptor of a spatially and directionally varying metric, and statistical approaches are less well developed, resulting in an increase in the quantity of data and complexity of the analysis [125,129,130].

With these difficulties noted, there are opportunities. Residual stress can often be reduced to an “average” or “maximum” residual stress within a part. Under such treatment, the application of many existing statistical approaches is appropriate. It is likely that the incorporation of modeling, including full FEA analysis with stress triaxiality and Lode parameter angle, will result in the most useful information that might then be analyzed using various statistical approaches. Conversely, a new statistical method has been recently leveraged that relies on building a Bayesian model from small sample builds to inform the in-plane deviation from distortion of new parts [306]. As most AM builds can be abstracted to simpler two-dimensional cross-sections, this model studied the distortion seen in differing cross-sections to inform a predictive model that could be applied to more geometrically complex shapes to see how much in-plane deviation would be found in final builds. This method is limited to individual cross-section analyses, so distortion in the build direction can go unseen but can be a powerful tool when paired with other techniques. In principle, if the training data are large enough, eventually there could be a machine learning algorithm made that takes in that data and suggests how the part’s geometry should be altered ahead of time so that the distortions are either minimized or lead to correct geometry in the final build.

### 3.5. Mechanical Properties

One of the important performance metrics for metallic parts made using fusion-based AM processes is the mechanical properties. Metal AM parts often have a vastly different material state and therefore different properties than wrought or cast parts. Some of the important mechanical properties are the (i) tensile properties, including yield strength, ultimate tensile strength, and ductility; (ii) fatigue properties, which are related to both the rough surfaces produced by AM processes as well as the residual stress; and (iii) fracture toughness.

Measuring the mechanical properties of metal AM samples is commonplace in materials characterization. The ex situ testing of AM samples can be conducted to obtain elastic and plastic properties, strength, hardness, toughness, and impact resistance. There are many standards for measuring the mechanical properties of metal samples with a wide range of instruments [307,308,309,310,311,312,313,314,315]. Most classical testing methods are destructive or alter the sample in some way, so most measurement techniques are not suitable for in situ monitoring.

#### 3.5.1. Monitoring of Mechanical Properties

A primary objective in the manufacturing of any arbitrary part is that the mechanical properties meet the design properties. Given the complexity and multi-scale nature of the process, it may be necessary to use a variety of in situ monitoring techniques to develop sufficient information to predict properties. Most of these systems monitor an aspect of the build that will affect the final properties (e.g., the melt pool stability, thermal gradients, defects, feedstock, and composition). Ultimately, monitoring information should be used to inform models to predict mechanical properties. Intriguingly, some mechanical properties would be possible to monitor during a build. For example, acoustic methods may be used to measure elastic modulus. However, even the use of acoustics for determining elasticity is not without difficulties, as the existence of thermal gradients during deposition necessarily means the elastic properties vary significantly throughout the part and are time-dependent. Similarly, hybrid additive/subtractive approaches have the potential to infer other mechanical properties by measuring machining forces [135,136]. However, this too is likely to be, at best, a time- and temperature-dependent surrogate measurement technique. Thus, in the main, there are no techniques to directly measure properties during deposition (Table 3).

#### 3.5.2. Modeling of Mechanical Properties

As noted previously, research from the welding community has provided a critical foundation for many models, including, in some cases, the mechanical behavior. While these models are generally empirical in nature, the fact remains that the mechanical properties often important to welding are also important to AM applications, such as yield and tensile strength, ductility, and impact toughness. Many of these models are based on the microstructure of the samples. These models are generally highly dependent on the material. For example, duplex stainless steel’s impact toughness depends on the phase fraction of austenite, which is dependent on the thermal history [288,316]. Other microstructural features influencing properties are composition, inclusion content, and acicular ferrite content in high-strength low alloy (HSLA) steels [276]. Similar to welding research, empirical relationships have been investigated for AM materials, such as Ti-6Al-4V [138]. These relations often consider the composition and microstructure of each material class.

The finite element method (FEM) has emerged as a preferred method for modeling solid mechanics. Therefore, many codes developed for predicting and modeling mechanical properties are based on FEM. One type of FEM that is prominent to local properties is crystal plasticity FEM (CPFE). CPFE uses slip as the primary mechanism for stress compensation, taking the form of dislocations. CPFE has been applied to many different microstructures and materials, and there are a variety of constitutive models that can be used in CPFE [317].

As a part of ExaAM, two codes, developed specifically for use in AM, are ExaConstit and Diablo [318,319]. ExaConstit is a crystal plasticity FEM code using a Newton–Raphson scheme. ExaConstit takes the microstructure data from the codes mentioned in Section 3.3 and thermal history data from the codes mentioned in Section 2.2. Diablo is a code intended to be the first step in the ExaAM workflow and provides the initial thermal history with additions of various constitutive models for mechanical behavior [210,305,319,320].

Since there are many features of the microstructure spanning many length scales that influence the properties of a material (grain size, porosity, phases, precipitates, and composition) [7,17,138], the integration of several models is necessary to gain an understanding of how the process influences many of these aspects. Model integration can help build a picture of the part to inform material property predictions [17,18,19,20,21,22,23]. This is, in essence, the reason for the advent of ICME, which assists with manufacturing parts with well-understood properties by taking several models that work on several length scales and can be used to create an informed simulation of a build and its properties. See Table 4 and Table 5.

#### 3.5.3. Statistics of Mechanical Property Models

Modeling and predicting mechanical properties, such as yield strength, often involves the creation of a constitutive equation, as has been the case for AM Ti-6Al-4V for example [25,26,47,138]. The variables that compose such constitutive equations are, by definition, variables associated with the material state, such as composition, texture, phase fractions, or dislocation densities, and continuing to improve monitoring efforts will only better inform models such as these. One novel approach to determining statistical information, such as “design allowables”, used these constitutive equations and their statistical distributions to predict, probabilistically, cumulative distribution functions, not breaking the constitutive model. This methodology, called distribution translation and rotation (DTR), has been shown to be effective in calibrating yield strength models for AM Ti-6Al-4V [25], and, as long as constitutive equations are made for their corresponding properties, could be extended for use in calibrating models for other mechanical properties built via other manufacturing methods.

## 4. Summary

This paper has reviewed the monitoring, modeling, and statistics associated with additive manufacturing of metallic systems. Throughout, the case has been made to consider multiple interrelationships, including (i) the importance of considering monitoring, modeling, and statistics concurrently; (ii) the importance of considering additive manufacturing from the perspective of both the process and the material state; and, (iii) the complex interdependencies that exist between and among aspects of the process (e.g., energy, feedstock, plume) and material state (e.g., composition, phase fraction). The three pillars of this paper—monitoring, modeling, and statistical analysis—are summarized briefly below.

Monitoring can be used to (i) help determine the presence of defects that would make the part unusable for operating conditions, (ii) provide data to optimize future parts, and (iii) provide information to create models that represent a specific aspect or characteristic of the part. By carefully considering the signals that may be naturally generated from the location being monitored, one can not only determine which technique is most appropriate to deploy but also suggest integrating techniques that are either used in other manufacturing sectors or new techniques entirely. New computational techniques used for monitoring in AM, based on machine learning or genetic algorithms, are proving to be valuable tools to analyze large data sets and provide quality assessments of the builds as well as inform statistical models. While some monitoring techniques have a level of maturity that can be easily applied to both laboratory and commercial systems (e.g., optical monitoring of melt pool and defects, or heat flow), others are considered emerging technologies (e.g., feedstock or distortion-focused monitoring), or only suitable for specific experiments and development efforts (e.g., synchrotron).

Modeling offers a way to understand the complex mechanisms that exist within AM processes. There are many models that have been used for AM. The first models that were created that are applicable to AM are moving heat source models. These have increased in complexity over the years and now can be extremely complex and thorough finite element models. The other part of AM processing that is modeled is the melt pool to understand how the energy source interacts with the feedstock and base material. These processing models are useful to understand how processing influences the material state. Therefore, to understand the material state better, models have been created that demonstrate the formation of defects, compositional variations, microstructure, residual stress, and distortion. Understanding how defects form from melt pool interactions and thermal histories that result in high residual stress is an important part of AM research, and models can assist with this research. Microstructure is influenced by thermal history. Residual stresses and distortion have been consistently researched. However, more research is needed to create better-informed models for future builds. Commercial software is available that models residual stress and distortion, especially for production manufacturing. Modeling is often performed with the intention that the information gathered can be used to understand the properties and performance of the material. Therefore, with validation, these models are useful to understand how properties are impacted, leading to the leverage of integrated computational materials engineering (ICME) to create well-informed builds quickly.

The statistical treatment of data, including the emerging in situ monitoring data associated with AM processes, is not only necessary to better understand the process and develop a fundamental understanding of the physics in operation but is also improved by integrating data into and from models. The process of better collection and better use of collected data creates a positive feedback loop where improved monitoring techniques help improve models, and improved models suggest what else needs monitoring. Data from monitoring processes can be used to better understand the system, as well as establish statistical methods such as confidence intervals to estimate how closely the monitoring data could be representative of the ground truth. As the real-time data analysis of monitoring increases, the use of machine learning to identify failed prints prematurely presents itself as both a time and material cost savings. For the modeling side, statistics can be used to bolster mathematical models to calibrate mechanical properties models of additively manufactured parts. The efforts behind model-informed qualification are pointing the way to approaches to accelerate the adoption of AM processes to manufacture parts for a variety of applications.

Each of these different threads has been treated relative to the process and the material state. However, there are only a few large research activities that seek to integrate subject matter expertise across the three domains covered by this paper. This gap arises largely due to the competition between requisite technical depth to advance each discrete approach (e.g., new sensor packages integrated into physical systems, computational algorithms, and physical architecture for on-board modeling incorporating real-time signals, accelerating AI/ML approaches) and the need to think broadly about the fundamental interdisciplinary nature of the problem. New research will be achieved by teams containing subject matter expertise that is only possible by working across research organizations.

## Figures and Tables

**Table 1:** Summary of state of the art on monitoring different variables and build characteristics of additive manufacturing methods.

AM Method	Variable/Characteristic Monitored							
	Feedstock	Processing Parameters	Melt Pool	Chemistry	Defects	Residual Stress and Distortion	Microstructure and Texture	Mechanical Properties
Laser PBF	A	B	A	B	A	A	B	B
Electron Beam PBF	A	B	A	B	A	A	X	X
Laser Powder DED	B	B	B	B	A	A	B	X
Laser Wire DED	X	B	B	X	X	B	X	X
Electron Beam Wire DED	B	B	B	X	A	B	X	X
WAAM	B	A	A	X	A	A	B	B

Note: A = Commonly studied, B = Limited research, X = No publications.

**Table 2:** Summary of state of the art on modeling different variables and build characteristics of additive manufacturing methods.

AM Method	Variable/Characteristic Modeled							
	Feedstock	Processing Parameters	Melt Pool	Chemistry	Defects	Residual Stress and Distortion	Microstructure and Texture	Mechanical Properties
Laser PBF	A	A	A	B	A	A	A	A
Electron Beam PBF	A	A	A	B	A	A	A	A
Laser Powder DED	B	A	A	B	B	B	B	A
Laser Wire DED	B	A	B	B	B	A	B	B
Electron Beam Wire DED	B	B	B	B	B	A	A	B
WAAM	B	A	A	B	B	A	A	A

Note: A = Commonly studied, B = Limited research.

**Table 3:** List of common monitoring techniques and the available data coming from these techniques.

Label	Description	Monitoring Technique *	References	Data Availability **	References
a	Feedstock	PBF_1_, DED_2_, and WAAM_2_: optical monitoring, compositional measurements	[34,35,36,37,42,44]	XX	[50,51,52]
b	Energy source	Laser_6_; electron beam_5_; wire arc_1,3_: multi-sensor electrical monitoring systems Heat flow_1_: NIR, thermal, and thermocouple	[53,54,55,56,57,58,59,60]	XXX	[61,62,63,64,65]
c	Melt pool	Melt pool_1,3_: optical, thermal, X-ray Vapor plume_1,4_: optical and chemical Spatter_1,4_: optical and thermal	[66,67,68,69,70,71,72,73,74,75,76,77,78,79,80,81,82,83,84,85,86,87,88,89,90,91]	XXX	[92,93,94,95]
d	Defects	Optical_1,3_, Thermal_1,3_, X-ray_1,3_, Acoustic_1,3_	[90,96,97,98,99,100,101,102,103,104,105]	XX	[63,106,107]
e	Compositional distribution	LIBS-based systems on vapor plume_2_, ultrasonic/acoustic_6_	[82,84,108,109,110]	X	[111,112]
f	Microstructure	IR_2_ and ultrasonic_2_	[113,114,115,116,117,118]	XX	[111,115]
g	Residual Stress and Distortion	Residual stress_2_: ultrasonic Distortion_1,3_: optical/DIC and displacement sensor	[119,120,121,122,123,124,125,126,127,128]	X	[125,129,130]
h	Mechanical Properties	Machining forces_5_, ultrasonic_5,6_, or acoustic_6_	[131,132,133,134,135,136,137]	X	[25,138]

* The availability of monitoring a parameter or characteristic is represented by 1—common in laboratory scale, 2—limited in laboratory scale, 3—common in commercial scale, 4—limited in commercial scale, 5—limited research, 6—potential applications but not commonly researched. **: The X-XX-XXX denotes a ranking of the amount of data that can be gathered from in situ monitoring techniques: X—limited data gathered, XX—medium amount of data gathered, XXX—large amounts of data gathered.

## Data Availability

No new data were created or analyzed in this study. Data sharing is not applicable to this article.
